# *ANRIL* rs4977574 Gene Polymorphism in Women with Recurrent Pregnancy Loss

**DOI:** 10.3390/jcm12185944

**Published:** 2023-09-13

**Authors:** Panagiotis Cherouveim, Despoina Mavrogianni, Eirini Drakaki, Anastasios Potiris, Athanasios Zikopoulos, Myrto Papamentzelopoulou, Konstantina Kouvoutsaki, Nikolaos Machairiotis, Theodoros Karampitsakos, Chara Skentou, Ekaterini Domali, Nikolaos Vrachnis, Peter Drakakis, Sofoklis Stavros

**Affiliations:** 1 Division of Reproductive Endocrinology and Infertility, Obstetrics and Gynecology, Massachusetts General Hospital, Harvard Medical School, Boston, MA 02114, USA; pcherouveim@mgh.harvard.edu; 2 First Department of Obstetrics and Gynecology, Alexandra Hospital, Medical School of the National and Kapodistrian University of Athens, 11528 Athens, Greece; dmavrogianni@med.uoa.gr (D.M.); eirinidrak@med.uoa.gr (E.D.); mpntua@yahoo.gr (M.P.); konskouv@med.uoa.gr (K.K.); kdomali@med.uoa.gr (E.D.); pdrakakis@med.uoa.gr (P.D.); 3 Third Department of Obstetrics and Gynecology, University General Hospital “ATTIKON”, Medical School of the National and Kapodistrian University of Athens, 12462 Athens, Greece; nikolaosmachairiotis@gmail.com (N.M.); theokarampitsakos@hotmail.com (T.K.); nvrachnis@med.uoa.gr (N.V.); sfstavrou@med.uoa.gr (S.S.); 4 Department of Obstetrics and Gynecology, Royal Cornwall Hospital, Treliske, Truro TR1 3LQ, UK; thanzik92@gmail.com; 5 Department of Obstetrics and Gynecology, Medical School of the University of Ioannina, 45110 Ioannina, Greece; haraskentou@gmail.com

**Keywords:** *ANRIL*, recurrent pregnancy loss, pregnancy loss, polymorphism, rs4977574

## Abstract

Background: *ANRIL* rs4977574 gene polymorphism has been associated with arterial thrombosis and cardiovascular disease development. *ANRIL* rs4977574 gene polymorphism could also be associated with recurrent pregnancy loss (RPL) since there is increasing evidence in favor of a potential shared pathophysiological mechanism with cardiovascular disease, potentially through arterial thrombosis. This study’s goal is to investigate the differences in *ANRIL* rs4977574 gene polymorphism between women with and without RPL, if any, as well as a potential association with the number of pregnancy losses. Methods: DNA was isolated from peripheral blood samples, and the sequence containing the polymorphism of interest was amplified with PCR. Results were visualized under UV light following electrophoresis in 3% agarose gel with ethidium bromide. *ANRIL* rs4977574 (A>G) prevalence was compared between 56 women with and 69 without RPL. Results were adjusted for women’s age and BMI, while a stratified analysis was performed according to number of pregnancy losses. Results: Allele A was significantly more prevalent in the control group compared to RPL women [31 (44.9%) vs. 14 (25%), *p* = 0.021]. Although not reaching statistical significance, a gradually decreasing prevalence of allele A with an increasing number of pregnancy losses was observed [31 (44.9%) in control, eight (30.7%) with two, six (23.1%) with three, and 0 (0.0%) with four pregnancy losses, *p* = 0.078]. Results were also similar following adjustment. Conclusions: This is the first study that demonstrates an association between RPL presence and *ANRIL* rs4977574 gene polymorphism (lower prevalence of allele A), while a difference according to the number of pregnancy losses cannot be excluded.

## 1. Introduction

Recurrent pregnancy loss (RPL) affects approximately 1–4% of women attempting pregnancy in Europe and the United States [1,2]. Despite its wide prevalence, shedding light on RPL pathophysiology and risk factors remains a challenge.

Many factors could interfere with the complex process of embryo–endometrium interaction and lead to pregnancy loss [3,4]. Amongst them, the role of vascular thrombosis in RPL pathophysiology has been recognized for more than 25 years [5]. Pregnancy, being a prothrombotic state, favors blood coagulation, which in turn could lead to pregnancy loss or other placenta-mediated complications [6]. Arterial thrombosis might have a more predominant role in RPL pathophysiology compared to venous thrombosis. Previous data suggest that even in diagnosed cases of venous thromboembolic disease, there is no clear benefit of a diagnostic workup and treatment [7]. On the other hand, many conditions leading to arterial thromboses have been linked to RPL. Lupus anticoagulant or anticardiolipin antibodies have been associated with arterial thromboses and may increase the risk of RPL [8]. The European Society of Human Reproduction and Embryology (ESHRE) and the American Society for Reproductive Medicine (ASRM) recommend investigating the presence of such antibodies in women with RPL, while they do not recommend investigating potential inherited thrombophilias [3,4]. Finally, a recent multicenter study found a correlation between pregnancy loss and cardiovascular risk [9], implying a shared underlying pathophysiological mechanism, at least partially, between the two disease processes.

A non-coding region in chromosome 9 (9p21) containing the Antisense Non-coding RNA in the INK4 Locus the CDKN2B antisense RNA 1 gene (*CDKN2B-AS1* or *ANRIL*) was discovered following Genome-Wide Association Studies (GWAS) that investigated the genetic basis of atheromatosis [10]. The *ANRIL* gene can form at least 20 circular or linear transcripts through alternative splicing [11,12,13], and its discovery remains very promising with many disease associations and implications [14,15,16,17,18,19,20,21]. One of its most prominent correlations seems to be with cardiovascular disease [10,14]. Ever since it was first described, there has been a body of steadily increasing evidence suggesting the critical role of *ANRIL* in the formation and progression of atheromatous plaques through vascular endothelial, smooth muscular, and mononuclear cells [22,23,24,25]. More specifically, rs4977574 polymorphism has been associated with atheromatosis in various arterial locations [26,27,28], leading to blood flow impediment to the heart [29,30] or the brain [31] and resulting in acute myocardial infarction [29,30] or ischemic stroke [31], respectively. Likewise, a similar event might lead to blood flow impediment in the uterine vessels, leading to the clinical manifestations of RPL. Congrains et al. reported an abnormal expression of *CDKN2A/B* and a decrease in cell growth when *ANRIL* was knocked down in cells of vascular smooth muscle [32]. According to Jarinova et al. and Congrains et al., the genetic variants of *ANRIL* influence atherosclerosis mechanisms such as thrombogenesis, vascular repair, and plaque stability by altering *ANRIL* expression and cell proliferation [32,33]. Xu Bing proposes that *ANRIL* changes the expression of the corresponding coding-related genes via mechanisms such as RNAi, gene silencing, or DNA methylation [30]. These data suggest that *ANRIL* rs4977574 gene polymorphism involvement in vascular disease and arterial thrombosis pathophysiology could provide a link to RPL development, further supporting the hypothesis of a shared underlying mechanism with cardiovascular disease.

One hypothesis would be *ANRIL* rs4977574 gene polymorphism being associated with arterial thrombosis, uterine blood flow restriction, and RPL manifestation. Consequently, the aim of our study is to investigate whether *ANRIL* rs4977574 gene polymorphism prevalence differs between women with or without RPL, as well as according to the number of pregnancy losses.

## 2. Materials and Methods

### 2.1. Study Design

This study included 125 women from the Alexandra University Hospital, First Department of Obstetrics and Gynecology, Medical School of the National and Kapodistrian University of Athens. The study protocol was approved by the scientific and ethics committee of the institution (Protocol Number 91970). Fifty-six women who had at least 2 prior pregnancy losses and who were aged less than 40 years old were included in the recurrent pregnancy loss (RPL) group. Sixty-nine women who had already had at least one livebirth without any prior pregnancy loss were included in the control group. Patient characteristics, such as age and BMI, were registered for the RPL group. The age and the BMI of the control group were matched to those in the RPL group. *ANRIL* rs4977574 gene polymorphism (A>G) prevalence was compared between the two groups. Data were not available for all partners and, consequently, were not included.

### 2.2. DNA Isolation and Detection of ANRIL rs4977574 Gene Polymorphism

Peripheral blood samples (2–3 mL) were collected, and DNA extraction was performed using the PureLink^®^ Genomic DNA Mini Kit (Invitrogen by Life Technologies, Waltham, MA, USA). Primers used for *ANRIL* polymorphism detection (rs4977574) were forward 5′-TTGAGGGTACATCAAAAGCATTCTATATCG-3′ and reverse 5′-TTTATTAGAGTGACTTGAACATCCCGT-3′. The conditions of the PCR were as follows: 95 °C for 1 min, 65 °C for 1 min, and 72 °C for 1 min. PCR products were visualized using agarose gel electrophoresis, and different fragments at 226 for allele G and 166 for allele A were detected.

### 2.3. Statistical Analysis

T-test, Mann–Whitney U-test, ANOVA, and Kruskal–Wallis H-test were used for numerical variables depending on each variable’s distribution and the number of groups compared. Chi-square test and Fisher exact test were used to compare categorical variables as appropriate. *ANRIL* rs4977574 gene polymorphism results were compared between controls and RPL, as well as after stratification of the RPL group according to the number of pregnancy losses. Odds ratios (ORs) and their respective 95% confidence intervals (CIs) were calculated using logistic regression to compare the odds of allele A prevalence between women with 2 and 3 pregnancy losses. The group of women with 4 pregnancy losses was not separately included in the regression models since it was comprised of only 4 women. OR results were adjusted for maternal age and BMI. Statistical significance was determined as a *p*-value < 0.05. Statistical analysis was performed using R (v4.1.0; R foundation for statistical programming).

## 3. Results

### 3.1. Baseline Characteristics

Table 1 summarizes baseline characteristics for the RPL group. In total, there were 56 women with RPL included in our analysis. The mean age was 35 for the women and 37.9 for their partners, while the mean women’s BMI was 23. Following stratification according to number of pregnancy losses, mean ages were 33.1, 36.7, and 35.8 years, their partner’s ages were 34.5, 40.7, and 36.5 years, and women’s BMI was 23.1, 22.8, and 24.7, among women with 2, 3, and 4 pregnancy losses, respectively. Both women (33.1 vs. 36.7 years, *p* = 0.019) and their partners (34.5 vs. 40.7 years, *p* = 0.027) with 2 pregnancy losses were younger compared to those with 3.

### 3.2. ANRIL rs4977574 Gene Polymorphism in RPL and Control Groups

**Figure 1:**
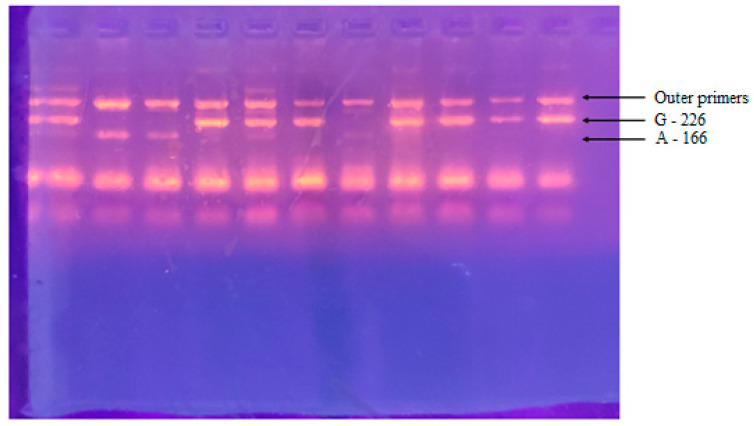
Agarose electrophoresis after PCR reaction. Lanes 1–11 show amplification of PCR products. A 226bp PCR product corresponds to a G allele. A 166bp PCR product corresponds to A allele. A 330bp PCR product is visualized after amplification with an outer primer pair.

**Figure 2:**
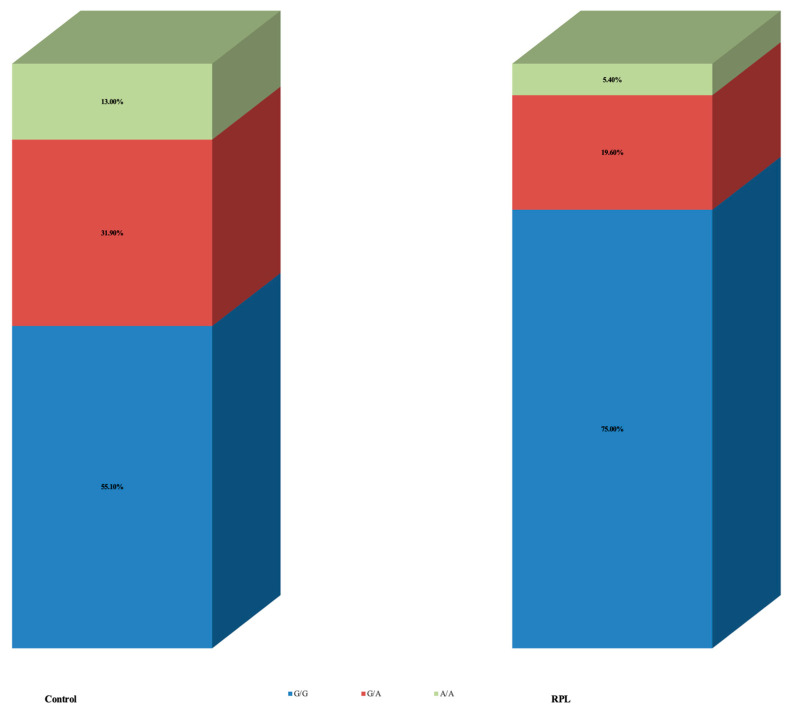
*ANRIL* (Antisense non-coding RNA in the INK4 Locus) rs4977574 polymorphism results between control and RPL (recurrent pregnancy loss).

The results presented were classified according to specific zones following electrophoresis (Figure 1). The *ANRIL* rs4977574 gene polymorphism results between the control and RPL groups are shown in Table 2. Women in the control group had allele A in a significantly higher frequency compared to RPL [31 (44.9%) control vs. 14 (25%) RPL, *p* = 0.021]. Of them, nine women (13.0%) in the control group and three (5.4%) in the RPL were homozygous for allele A (genotype A/A). When analysis was based on specific genotypes, the results did not reach statistical significance (*p* = 0.062) (Figure 2).

### 3.3. ANRIL rs4977574 Gene Polymorphism Depending on Number of Pregnancy Losses

**Figure 3:**
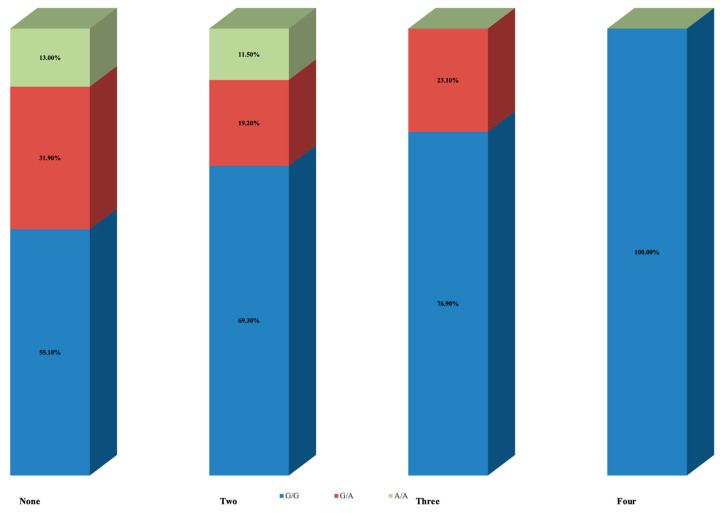
*ANRIL* (Antisense Non-coding RNA in the INK4 Locus) rs4977574 polymorphism results according to number of pregnancy losses.

Table 3 summarizes the results following stratification according to the number of pregnancy losses. The first group (None) represents the control group, which included women with no pregnancy losses. Even though results did not reach statistical significance (*p* = 0.078), there was a pattern of gradually decreased prevalence of allele A with an increasing number of pregnancy losses. Thirty-one (44.9%) of women in the control group had at least one allele A, eight (30.7%) with two pregnancy losses, six (23.1%) with three, and none with four pregnancy losses. Of them, nine women (13.0%) in the control group, three (11.5%) with two pregnancy losses, and none with three were homozygous for allele A (genotype A/A) (Figure 3).

### 3.4. ANRIL rs4977574 Gene Polymorphism in Women with 2 and 3 Pregnancy Losses

Finally, OR for allele A presence between women with 2 and 3 pregnancy losses was not significant neither before [OR (95%CI): 0.68(0.20–2.32), *p* = 0.533], nor after adjustment [adjOR(95%CI): 0.54(0.14–2.13), *p* = 0.375].

## 4. Discussion

Our study investigated whether *ANRIL* rs4977574 gene polymorphism prevalence differs between women with or without RPL, as well as according to the number of pregnancy losses. The results suggest that allele A was more prevalent among women with RPL compared to those without, with a high percentage of heterozygosity (G/A) in both groups. After stratification according to the number of pregnancy losses, even though the results did not reach statistical significance, allele A prevalence decreased with an increasing number of pregnancy losses both before and after accounting for women’s age and BMI. Thus, our results suggest that there might be an association of *ANRIL* rs4977574 gene polymorphism with RPL diagnosis, while an association with the number of pregnancy losses could not be excluded.

Since there are no studies investigating polymorphism rs4977574 in the setting of RPL, we will focus on the literature related to cardiovascular disease and potential molecular mechanisms implicated in the formation, rupture, and thrombosis of the atheromatous plaque. These processes are partially mediated by *ANRIL* gene expression in vascular endothelial, smooth muscle, and mononuclear cells [22,23,24,25]. *ANRIL* mediates inflammatory response and enhances vascular endothelial damage through TNF-α-NF-κB-*ANRIL*/YY1-IL6/8 pathway [34], caspase recruitment domain-containing protein 8 [35], and vascular endothelial growth factor [36] expression increase. Moreover, *ANRIL* has been associated with abnormal proliferation, migration, aging, and apoptosis of vascular smooth muscle cells [32,37,38,39]. *ANRIL* has also been found to regulate cyclin-dependent kinase inhibitor 2A and 2B expression, which have a major role in the regulation of the cell cycle, apoptosis, and cell aging [37,40]. Finally, Alu elements, a family of short interspersed repeat elements, are associated with atheromatosis by facilitating mononuclear cell adhesion and proliferation [41] and have been found to be functionally related to *ANRIL* in knock-out studies [42,43]. Taken together, this evidence highlights the role of *ANRIL* in many aspects of atheromatosis and cardiovascular disease pathophysiology, which might also be associated with RPL.

To the best of our knowledge, there are no published data about *ANRIL* gene polymorphism prevalence in women with RPL. Therefore, we provide the first preliminary data on *ANRIL* rs4977574 gene polymorphism among women with RPL as part of a novel investigation of the common pathophysiology shared between RPL and cardiovascular disease. We found a significant association between *ANRIL* rs4977574 gene polymorphism and RPL diagnosis. Two recent metanalyses have found an association between *ANRIL* rs4977574 gene polymorphism and a higher risk for coronary artery disease [30,44]. Both studies concluded that allele G was associated with increased risk [30,44]. Similarly, our results suggest a higher prevalence of allele G in women with RPL. Taking into consideration the higher probability of cardiovascular disease development later in life among women with pregnancy loss [9], these results further support the hypothesis of a shared underlying pathophysiological mechanism that contributes to both conditions.

Formation and progression of the atheromatous plaque might explain, at least partially, the basis of the shared underlying mechanism. *ANRIL* rs4977574 gene polymorphism has been associated with atheromatosis in coronary [27] and carotid arteries [26,28], which is probably independent of hypertension [45]. Additionally, consumption of vegetables, wine [46], and smoking [47] seem to further modify the risk for cardiovascular disease associated with the polymorphism. The ultimate result is atheromatous plaque rupture and vessel thrombosis [27]. Depending on the location of the affected vessel, there is the respective clinical manifestation of acute myocardial infarction [29,30] or ischemic stroke [31]. Therefore, one potential explanation would be that the presence of allele G in rs4977574 increases the risk for atheromatosis [26,27,28] and, thus, arterial thrombosis, leading to RPL.

Our study is the first to investigate *ANRIL* gene polymorphisms in women with RPL. The number of pregnancy losses, age, and BMI have been found to be correlated to RPL [2,48] and adjusting for them enhances the validity of our results. Nevertheless, there were no available demographic data for women or their partners in the control group, restricting relevant analyses. Additionally, we do not have consistent data available on other factors that might also contribute to RPL development, such as the presence of thrombophilia, chromosomal abnormalities, or autoimmune diseases.

Future studies could aim at investigating the underlying molecular mechanisms that might explain a potential *ANRIL* and RPL correlation. In addition to utilizing next-generation sequencing, including more baseline demographic characteristics of the couples could help establish useful RPL biomarkers.

## 5. Conclusions

In summary, this study provides the first possible association between *ANRIL* gene polymorphism and recurrent abortions. Our results suggest that rs4977574 is associated with RPL prevalence, while an association with the number of pregnancy losses cannot be excluded. Although the mechanisms underlying the aforementioned association are not clarified, the present study proposes that a shared pathophysiological mechanism is possible for both RPL and cardiovascular disease, potentially through atheromatosis and arterial thrombosis as genetic variants of *ANRIL* influence atherosclerosis mechanisms such as thrombogenesis, vascular repair, and plaque stability by altering *ANRIL* expression and cell proliferation. Consequently, the studied polymorphism could be proposed as a possible biomarker of recurrent abortions, and additionally, future studies may focus on the genes interfering with *ANRIL* and also on genes in linkage disequilibrium with the studied polymorphism.

## Figures and Tables

**Table 1:** RPL group baseline characteristics.

Variable	All RPL	2 Losses	3 Losses	4 Losses	*p*-Value *
	(Ν = 56)	(Ν = 26)	(Ν = 26)	(Ν = 4)	
**Women’s age**					
***(years)***					
Mean (SD)	35.0 (5.7)	33.1 (5.6)	36.7 (5.2)	35.8 (7.3)	
Median (IQR)	35.0 (31.0, 40.0)	33.0 (30.0, 38.0)	38.0 (32.0, 41.0)	34.0 (30.0, 43.3)	0.019
**BMI**					
***(kg/m^2^)***					
Mean (SD)	23.0 (3.1)	23.1 (3.2)	22.8 (3.1)	24.7 (0.4)	
Median (IQR)	22.5 (20.3, 25.1)	22.7 (20.7, 24.2)	22.1 (20.2, 25.7)	24.7 (24.7, -)	0.914
**Partner’s age**					
***(years)***					
Mean (SD)	37.9 (5.8)	34.5 (3.7)	40.7 (6.7)	36.5 (0.7)	
Median (IQR)	37.0 (34.0, 40.0)	34.5 (31.8, 38.5)	37 (36, 46)	36.5 (36.0, -)	0.027

* 2 vs. 3 pregnancy losses; (-): not able to calculate. RPL: recurrent pregnancy loss; BMI: body mass index.

**Table 2:** *ANRIL* rs4977574 polymorphism results between control and RPL.

*ANRIL* rs4977574 Polymorphism	Controls (n = 69)	RPL (n = 56)	*p*-Value
G/G	38 (55.1%)	42 (75.0%)	
G/A	22 (31.9%)	11 (19.6%)	0.062, for genotype
A/A	9 (13.0%)	3 (5.4%)	0.021, for allele A

*ANRIL*: Antisense non-coding RNA in the INK4 Locus; RPL: recurrent pregnancy loss.

**Table 3:** *ANRIL* rs4977574 polymorphism results according to number of pregnancy losses.

*ANRIL* rs4977574 Polymorphism	None (n = 69)	Two (n = 26)	Three (n = 26)	Four (n = 4)	*p*-Value
G/G	38 (55.1%)	18 (69.3%)	20 (76.9%)	4 (100%)	
G/A	22 (31.9%)	5 (19.2%)	6 (23.1%)	0 (0.0%)	0.188, for genotype
A/A	9 (13.0%)	3 (11.5%)	0 (0.0%)	0 (0.0%)	0.078, for allele A

*ANRIL*: Antisense non-coding RNA in the INK4 Locus.

## Data Availability

Not applicable.
